# Effect modification by dietary patterns in the relationship between slow gait and incident depressive symptoms: a 6-year cohort study of older Japanese adults (NISSIN Project)

**DOI:** 10.3389/fnut.2025.1698581

**Published:** 2025-12-10

**Authors:** Wen Hao, Yi-fan Shan, Takashi Kimura, Shigekazu Ukawa, Hideki Ohira, Satoe Okabayashi, Kenji Wakai, Masahiko Ando, Akiko Tamakoshi

**Affiliations:** 1 School of Public Health, Zhejiang Chinese Medical University, Hangzhou, China; 2 Department of Public Health, Faculty of Medicine and Graduate School of Medicine, Hokkaido University, Sapporo, Japan; 3 First Affiliated Hospital of Zhengzhou University, Zhengzhou, China; 4 Osaka Metropolitan University Graduate School of Human Life and Ecology, Osaka, Japan; 5 Department of Psychology, Graduate School of Informatics, Nagoya University, Nagoya, Japan; 6 Agency for Health, Safety and Environment, Kyoto University, Kyoto, Japan; 7 Department of Preventive Medicine, Graduate School of Medicine, Nagoya University, Nagoya, Japan; 8 Center for Advanced Medicine and Clinical Research, Nagoya University Hospital, Nagoya, Japan

**Keywords:** gait speed, dietary pattern, depressive symptoms, older adults, effect modification

## Abstract

**Introduction:**

Slower gait speed is a well-established predictor of late-life depressive symptoms. Previous research suggests that diet can influence biological processes implicated in both gait decline and depression and may therefore serve as an effect modifier in their association. This study aimed to examine whether adherence to healthy dietary patterns modifies the relationship between gait speed and incident depressive symptoms in older adults.

**Methods:**

In this longitudinal observational cohort study, we analyzed data from 1,887 depression-free, community-dwelling adults aged 64–65 years, obtained from the New Integrated Suburban Seniority Investigation (NISSIN) Project in Japan. Gait speed was self-rated at baseline as fast, normal, or slow. Dietary intake was assessed using a validated food frequency questionnaire (FFQ), and principal component analysis was performed to identify three dietary patterns: vegetables, Fat and Meat, and Bread and Egg. Incident depressive symptoms were defined as having a 15-item Geriatric Depression Scale (GDS-15) score of ≥6 after a 6-year follow-up. Modified Poisson regression was used to estimate relative risks (RRs), and interactions were assessed on multiplicative and additive scales.

**Results:**

After 6 years, 12.5% of the participants developed depressive symptoms. Slow gait speed was significantly associated with a higher risk of depression (RR: 2.7; 95% CI: 1.6–4.6), while no dietary pattern was independently associated with depressive symptoms. This risk tended to be lower among slow walkers with higher adherence to a vegetable or Bread and Egg dietary pattern, although the interactions were not statistically significant. A significant negative interaction was found between slow gait and low adherence to a Fat and Meat diet on both additive (RERI = −2.5; 95% CI: −4.1 to −0.8) and multiplicative scales (Ratio of RRs: 0.3; 95% CI: 0.2–0.3).

**Discussion:**

Dietary patterns may influence the link between slow gait and depression in older adults. Notably, reduced adherence to Fat and Meat diets was associated with a lower overall risk. These findings support dietary improvement as a scalable mental health strategy for physically vulnerable older adults.

## 1 Introduction

Slower gait speed has been increasingly recognized as a critical early marker of various late-life health outcomes in older adults, including incident disability (1), dementia-related diseases (2, 3), and mortality (4). A growing body of evidence also indicates that reduced gait speed is a robust predictor of psychological outcomes, particularly depressive symptoms (5). For instance, a large-scale longitudinal study involving 72,359 participants found that gait speed predicts incident depression in middle-aged and older adults (6). These findings were further supported by a meta-analysis, which demonstrated that gait speed could predict the onset of depression across follow-up periods ranging from 12 months to 16 years (7). Due to the impact of slow gait on individuals' health and quality of life and its ability to predict future deterioration of health, research interest is shifting toward identifying potentially modifiable causes of gait speed decline and slow gait speed (8).

The prevailing view is that gait speed is not merely a functional output but reflects underlying neurophysiological processes, such as brain structural integrity, oxidative stress, and neuroinflammation, which are central to the onset of depression (9). Many of these biological pathways are, in turn, sensitive to a range of social (10) and lifestyle influences, with diet being among the most notable (11). Nutrients in diets are thought to provide neuroprotective, anti-inflammatory, and metabolic benefits that may counteract some of the adverse pathways linking poor mobility to mental health decline (12, 13). This overlap suggests that dietary habits might modify the psychological impact of slow gait speed.

Diet has emerged as a promising strategy for promoting both mental and physical health in aging populations (14, 15). Healthy dietary patterns, particularly those resembling the Mediterranean diet, which are rich in fruits, vegetables, whole grains, legumes, fish, and unsaturated fats, have been associated with improved mobility and lower levels of depressive symptoms (16–18). Given these findings, dietary habits may serve as a plausible effect modifier in the relationship between gait speed and depressive symptoms. However, to date, no study has explicitly examined whether adherence to a healthy diet can buffer or modify the psychological impact of slow gait speed in older adults.

Therefore, this study aims to investigate the potential effect modification by dietary quality in the association between gait speed and depressive symptoms in older adults. We hypothesize that older adults adhering to a healthy diet at baseline will exhibit a weaker longitudinal association between slower gait speed and depressive symptoms compared to their counterparts with poorer dietary habits. Understanding this potential interaction could provide valuable insights into individual-level risk stratification and inform future strategies to support mental health in aging populations.

## 2 Materials and methods

Data for this study were obtained from the New Integrated Suburban Seniority Investigation (NISSIN) Project, an ongoing, age-specific prospective cohort study conducted in Aichi Prefecture, Japan. Detailed information on the study design has been provided elsewhere (19). Briefly, between 1996 and 2005, all residents of Nisshin City who reached the age of 64 or 65 were continuously invited to participate in a free, comprehensive health checkup and to complete a self-administered questionnaire. Six years later, when participants reached the age of 70 or 71, they were invited for a follow-up health examination and questionnaire survey. For those who remained in the city but did not attend the follow-up health checkup, municipal public health nurses conducted home visits to complete the questionnaire survey.

### 2.1 Evaluation of depressive symptoms

Depressive symptoms were evaluated using the Japanese version of the 15-item Geriatric Depression Scale (GDS-15) at both baseline and follow-up. The GDS-15 consists of 15 questions, each with only “Yes” and “No” response options. A total score, ranging from 0 to 15, can be calculated, and higher scores indicate more depressive symptoms. Depressive symptoms were defined as a score of 6 or greater, which has been shown to have good sensitivity (0.973) and specificity (0.959) for detecting depressive symptoms in the Japanese population (20).

### 2.2 Dietary intake assessment

Dietary intake was recorded using a validated food frequency questionnaire (FFQ) that included 97 modern Japanese food items at baseline (21). The participants were asked to report their average intake frequency of these food items over the year prior to the survey. Food frequencies were measured on an incremental scale (< once/month, once/month, 2–3 times/month, once/week, 2–4 times/week, 5–6 times/week, once/day, 2–3 times/day, ≥4 times/day), and portion sizes were fixed for all foods except rice. A total of 20 food groups were categorized based on similarities among foods, including rice, bread, noodles, other cereals, potatoes, sugar, confectionery, oils and fats, nuts, beans, seafood, meat, eggs, dairy products, fruits, mushrooms, algae, green/yellow vegetables, other vegetables, and seasoning. We assessed dietary patterns based on the intake of major food groups (e.g., vegetables and fruits, oils and fats, bread, and dairy products), which are commonly consumed both at home and when eating out.

### 2.3 Subjective gait speed

Subjective gait speed was assessed at baseline using the self-administered questionnaire, which included the following question: “How do you feel about your walking speed during the past year?” The response options were “Fast,” “Normal,” and “Slow.”

### 2.4 Covariates

Sociodemographic characteristics were measured at baseline. Age, sex, educational background, smoking and drinking status, living arrangements, regular exercise habits, medical history, and social activity measures were included in the self-administered questionnaire. Height and weight were measured using professional-grade equipment. Body mass index (BMI) was calculated as weight in kilograms divided by height in meters squared. Medical history included the presence of hypertension, diabetes, hyperlipidemia, and cardiovascular diseases (CVDs). Participants were considered to have CVDs if their medical reports indicated any of the following conditions: myocardial infarction, angina pectoris, arrhythmia, other heart diseases, cerebral infarction, intracerebral hemorrhage, subarachnoid hemorrhage, or other cerebrovascular diseases. Functional activity was evaluated using the instrumental activities of daily living (I-ADL) subset of the Tokyo Metropolitan Institute of Gerontology Index of Competence. The participants were considered self-independent if they scored >11 (22).

### 2.5 Statistical methods

Demographic and clinical characteristics of the slow, normal, and fast walkers were summarized using frequencies and percentages, and the group differences were compared using chi-squared tests. We used the residual method to adjust the intake of food groups for total energy (23). Dietary patterns were determined using principal component analysis with varimax rotation based on 20 energy-adjusted food groups. Food groups with factor loadings >0.5 were considered dominant contributors to dietary patterns, and those with loadings below ± 0.2 were not reported for better interpretation. We calculated the factor score for each dietary pattern by summing the products of food group consumption and their respective factor loadings, and a higher factor score indicated better adherence to this dietary pattern. Each factor score was then divided into tertiles, classifying the participants into low, moderate, and high adherence groups.

Modified Poisson regression models were used to estimate relative risks (RRs) and corresponding 95% confidence intervals (CIs) for progression to depressive symptoms across the gait speed groups, the dietary pattern tertiles, and the nine-category interaction term of gait speed and dietary pattern. We first presented RRs for each stratum of gait speed and dietary patterns using a single reference category, then presented RRs for gait speed within the strata of dietary patterns. High adherence to the dietary pattern and fast gait speed were set as the reference group. We evaluated effect modification by dietary patterns in the association between gait speed and depression by calculating both multiplicative (ratios of RR) and additive scales (relative excess risks due to interaction, RERI) as suggested by Knol and VanderWeele (24). The delta method described by Hosmer and Lemeshow was used for the estimation of CIs and *p-*values (25).

As the outcome of depressive symptoms was relatively common (>10% incidence), we used a modified Poisson regression model instead of a logistic model to avoid overestimation of odds ratios, which can occur with the latter model (26). The covariates that were adjusted in the model were as follows: sex, BMI (< 18.5, 18.5–25, and ≥25), educational background (junior high or lower, high school, college or higher), current drinking and smoking status (yes or no), living alone (yes or no), regular exercise (yes or no), I-ADL (>11 or ≤ 10), hypertension (yes or no), diabetes (yes or no), hyperlipidemia (yes or no), and cardiovascular diseases (CVDs; yes or no). Statistical significance was set at a *p-*value of < 0.05 (two-sided). Statistical analyses were performed using SAS 9.4 (SAS Institute Inc., Cary, NC, USA).

## 3 Results

**Figure 1:**
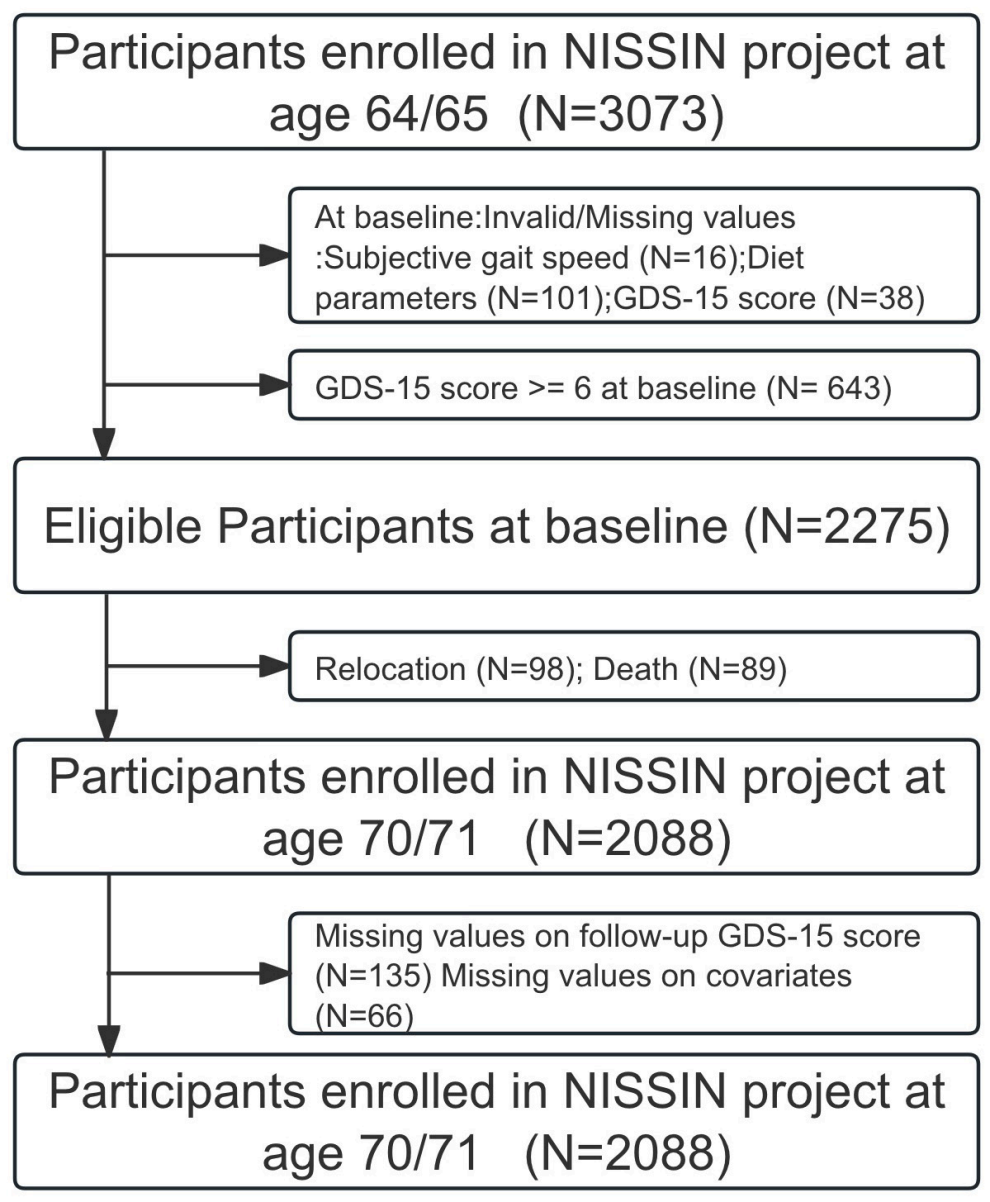
Study population.

A total of 3,073 participants took part in the baseline study. We first excluded participants with missing values for subjective gait speed (*N* = 16), dietary patterns (*N* = 101), or GDS-15 scores (*N* = 38) at baseline. To identify newly incident depressive symptoms, we then excluded those with baseline GDS-15 scores ≥6 (*N* = 643). During the 6-year follow-up, 98 participants relocated, and 89 of them died. We further excluded those with missing values for GDS-15 scores (*N* = 135) and covariates (*N* = 66) at follow-up. Overall, 1,887 valid participants were included in the analyses. After 6 years, 236 participants (12.53%) developed depressive symptoms (Figure 1).

Principal component analysis yielded three dietary patterns. The vegetable dietary pattern included different kinds of vegetables, mushrooms, algae, and fruits; the Fat and Meat dietary pattern included the intake of oil, fat, and meat; and the Bread and Egg dietary pattern was characterized by high factor loadings of dairy products and bread and a low intake of rice (Table 1).

**Table 1:** Rotated factor loadings for each dietary pattern.

**Food groups**	**Vegetables**	**Fat and Meat**	**Bread and Egg**
Vegetables	**0.86**		
Other vegetables	**0.73**	0.19	
Green/Yellow vegetables	**0.69**		
Mushrooms	**0.58**		
Algae	**0.57**		
Fruits	**0.54**		0.34
Beans	0.47		
Seafood	0.43	0.24	−0.20
Other grains		**0.79**	
Oils and fats		**0.74**	0.27
Meat		**0.70**	
Root and tubers	0.39	0.48	
Seasoning	0.36	0.41	0.26
Rice	−0.37	−0.41	–**0.69**
Bread			**0.58**
Eggs			**0.50**
Sugar		0.39	0.49
Dairy products		−0.29	0.47
Noodles	−0.24		0.35
Confectionery			0.27
Nuts	0.28		

Bold values indicate factor loadings greater than 0.5.

Table 2 presents the baseline demographic and clinical characteristics by gait speed. Compared to the fast walkers, the slow walkers were more likely to be overweight, have a lower educational level, be current smokers, drink less alcohol, not engage in regular exercise, and have a history of diabetes and CVD. In addition, the slow walkers tended to consume fewer vegetables than the fast walkers.

**Table 2:** Demographics of the participants by subjective gait speed at baseline.

**Personal characteristics**	**Total**	**Baseline gait speed**			***P* Value**
		**Slow**	**Normal**	**Fast**	
Number	1,887	184	1,476	293	
**Sex**, ***N*** **(%)**				
Male	972 (51.5)	83 (45.9)	747 (52.5)	142 (50.4)	0.226
Female	915 (48.5)	98 (54.1)	677 (47.5)	140 (49.7)	
**BMI**, ***N*** **(%)**				
< 18.5	90 (4.8)	5 (2.8)	70 (4.9)	15 (5.3)	< 0.001
18.5–25	1,381 (73.2)	118 (65.2)	1,031 (72.4)	232 (82.3)	
≥25	416 (22.1)	58 (32.0)	323 (22.7)	35 (12.4)	
**Educational background**, ***N*** **(%)**				
Junior high and lower	523 (27.7)	58 (32.0)	408 (28.7)	57 (20.2)	0.019
High school	870 (46.1)	81 (44.75)	653 (45.9)	136 (48.2)	
93.6-2.2,-1.3498ptCollege and higher	494 (26.2)	42 (23.2)	363 (25.5)	89 (31.6)	
**Current drinker*****, N*** **(%)**	**880 (46.6)**	**67 (37.0)**	**668 (46.9)**	**145 (51.4)**	**0.009**
**Current smoker**, ***N*** **(%)**	**311 (16.5)**	**30 (16.6)**	**252 (17.7)**	**29 (10.3)**	**0.009**
**Living alone**, ***N*** **(%)**	**63 (3.3)**	**5 (2.7)**	**45 (3.1)**	**13 (4.4)**	**0.419**
**Regular exercise**, ***N*** **(%)**	**1,029 (54.5)**	**61 (33.7)**	**760 (53.4)**	**208 (73.4)**	<**0.001**
**IADL** >**11**, ***N*** **(%)**	**1,738 (92.1)**	**163 (90.1)**	**1,317 (92.5)**	**258 (91.5)**	**0.478**
**Baseline medical history**, ***N*** **(%)**				
Diabetes	144 (7.6)	24 (13.3)	99 (7.0)	21 (7.5)	0.011
Hyperlipidemia	257 (13.6)	27 (14.9)	195 (13.7)	35 (12.4)	0.735
Hypertension	448 (23.7)	50 (27.6)	345 (24.2)	53 (18.8)	0.064
CVD	129 (6.8)	15 (8.3)	108 (7.6)	6 (2.1)	0.003
**Vegetables**, ***N*** **(%)**				
Low	629 (33.3)	68 (37.6)	483 (33.9)	78 (27.7)	0.003
Moderate	629 (33.3)	54 (29.8)	490 (34.4)	85 (30.1)	
High	629 (33.3)	59 (32.6)	451 (31.7)	119 (42.2)	
**Fat and Meat**, ***N*** **(%)**				
Low	629 (33.3)	45 (24.9)	481 (33.8)	103 (36.5)	0.068
Moderate	629 (33.3)	74 (40.9)	469 (32.9)	86 (30.5)	
High	629 (33.3)	62 (34.3)	474 (33.3)	93 (33.0)	
**Bread and Egg**, ***N*** **(%)**				
Low	629 (33.3)	71 (39.2)	478 (33.6)	80 (28.4)	0.184
Moderate	629 (33.3)	57 (31.5)	473 (33.2)	99 (35.1)	
High	629 (33.3)	53 (29.3)	473 (33.2)	103 (36.5)	
**Depressive symptoms at 70**, ***N*** **(%)**	**236 (12.5)**	**39 (21.6)**	**179 (12.6)**	**18 (6.4)**	<**0.001**

N, number; BMI, body mass index; IADL, instrumental activities of daily living.

### 3.1 Associations of gait speed and dietary patterns with depressive symptoms

Compared to the fast walkers, the slow and normal walkers had significantly higher RRs for developing depressive symptoms after the 6-year follow-up. These associations were independent of lifestyle factors and medical history. In the fully adjusted models, the slow walkers had nearly a threefold increased risk of developing depressive symptoms (RR: 2.7, 95% CI: 1.6–4.6, *p* < 0.001), while normal walkers had nearly a twofold increased risk compared to the fast walkers (1.8, 1.1–2.9, *p* = 0.007). However, no significant associations were found between any of the three dietary patterns and depressive symptoms (Table 3).

**Table 3:** Associations between gait speed, dietary patterns, and depressive symptoms.

**Variables**	**Crude model**			**Adjusted model** ^*^		
	**RR**	**Lower CI**	**Upper CI**	**RR**	**Lower CI**	**Upper CI**
**Subjective gait speed**						
Fast	1			1		
Normal	2.0	1.2	3.1	1.8	1.1	2.9
Slow	3.4	2.0	5.7	2.7	1.6	4.6
**Vegetables pattern**						
High	1			1		
Moderate	1.2	0.9	1.6	1.2	0.9	1.7
Low	1.7	0.9	1.7	1.3	0.9	1.7
**Fat and Meat pattern**						
High	1			1		
Moderate	0.8	0.6	1.0	0.8	0.6	1.1
Low	1.0	0.8	1.3	1.0	0.8	1.4
**Bread and Egg pattern**						
High	1			1		
Moderate	1.2	0.9	1.6	1.1	0.8	1.5
Low	1.0	0.8	1.4	1.0	0.7	1.3

^*^The models were adjusted for sex, BMI, educational background, living alone, smoking and drinking habits, exercise habits, IADL score, hypertension status, diabetes status, hyperlipidemia status, and CVD status.

### 3.2 Effect modification by dietary pattern in the association between gait speed and depressive symptoms

**Figure 2:**
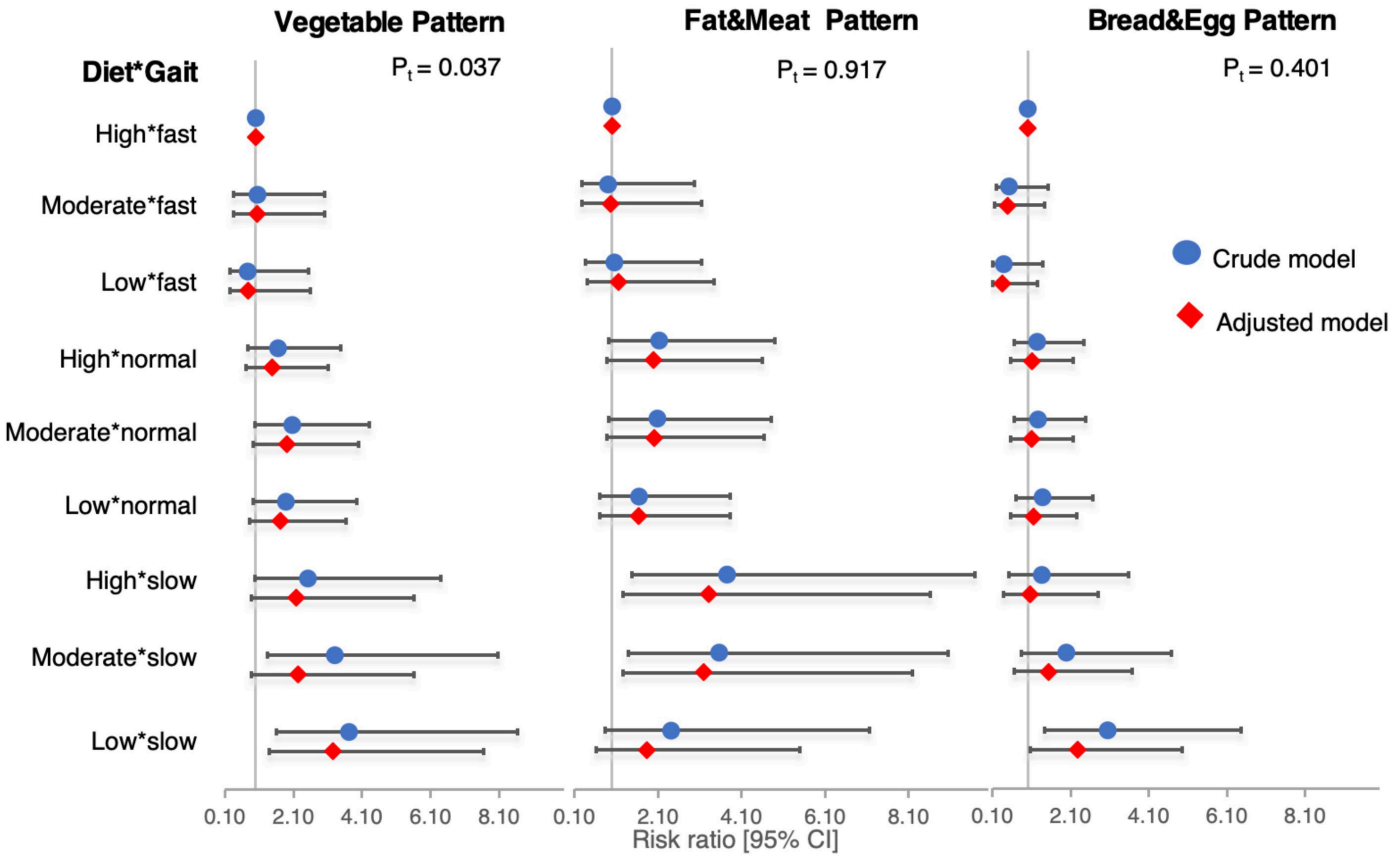
Forest plot of effect modification of dietary pattern on association between gait speed and depressive symptoms.

Compared to the participants who walked fast and had low vegetable adherence (reference), the slow walkers with low vegetable adherence showed a threefold higher risk of depressive symptoms (3.3, 1.4–7.7, *p* = 0.01). In contrast, the risk among the slow walkers with moderate or high vegetable adherence was not significant. A significant dose–response trend across the nine gait–diet combinations was observed (*p* for trend = 0.037). The additive interaction index (RERI = +1.3, 95% CI −0.5 to 3.2) and the multiplicative index (RR_ratio = 1.6, 95% CI: 0.3–9.8) suggested a potential positive interaction, although CIs marginally included the null (Table 4, Figure 2).

**Table 4:** Modification of the effect of gait speed on depressive symptoms by adherence to the Vegetable dietary pattern.

	**Adherence to the vegetable dietary pattern**						**RRs (95%CI) for moderate adherence within gait speed strata**	**RRs (95%CI) for low adherence within gait speed strata**
	**High**		**Moderate**		**Low**			
	***N*** **cases/controls**	**RR (95% CI)**	***N*** **cases/controls**	**RR (95% CI)**	***N*** **cases/controls**	**RR (95% CI)**		
**Gait speed**								
Fast	8/119	Reference	6/85	1.1 (0.4–3.1), *p* = 0.95	4/78	0.8 (0.3–2.7), *p* = 0.69	1.4 (0.5–3.8), *p* = 0.63	1.2 (0.4–3.5), *p* = 0.78
Normal	50/451	1.5 (0.7–3.2), *p* = 0.31	68/490	1.9 (0.9–4.1), *p* = 0.09	61/483	1.8 (0.9–3.7), *p* = 0.17	1.3 (0.9–1.9), *p* = 0.14	1.2 (0.8–1.7), *p* = 0.49
Slow	10/59	2.2 (0.9–5.6), *p* = 0.11	12/54	2.3 (0.9–5.7), *p* = 0.09	17/68	3.3 (1.4–7.7), *p* = 0.01	1.1 (0.5–2.4), *p* = 0.85	1.5 (0.7–3.1), *p* = 0.37
RRs (95%CI) for normal gait within dietary pattern strata		1.5 (0.8–3.1), *p* = 0.29		1.9 (0.9–4.1), *p* = 0.14		2.0 (0.8–5.4), *p* = 0.19		
RRs (95%CI) for slow gait within dietary pattern strata		2.3 (1–5.5), *p* = 0.08		2.3 (0.9–5.7), *p* = 0.09		3.6 (1.3–10.3), *p* = 0.03		
Measure of interaction on the additive scale: RERI_lowandslow_ (95%CI)			1.3 (−0.5–3.2); *p* = 0.15			
Measure of interaction on the multiplicative scale: Ratio of RR_lowandslow_ (95%CI)			1.6 (0.3–9.8); *p* = 0.63			

RRs were adjusted for sex, BMI, educational background, living alone, smoking and drinking habits, exercise habits, IADL score, hypertension status, diabetes status, hyperlipidemia status, and CVD status.

A contrary trend was observed in the gait–Fat and Meat combination, in which we found that the slow walkers with high (3.3, 1.3–8.6, *p* = 0.01) and moderate (3.2, 1.2–8.1, *p* = 0.02) adherence to the Fat and Meat dietary pattern had significantly higher risks of developing depressive symptoms compared to those with both high adherence to the Fat and Meat dietary pattern and fast gait speed (1.8, 0.6–5.5, *p* = 0.28). In addition, we observed a significant negative interaction between slow gait and low fat-meat adherence on the association with incident depressive symptoms, with RERI = −2.5 (−4.1 to −0.8, *p* < 0.01) and a ratio of RRs = 0.3 (0.2–0.3, *p* < 0.01), indicating that the combined effect of slow gait and low adherence to the Fat and Meat diet on depressive symptoms was significantly less than the sum of their individual effects (Table 5, Figure 2).

**Table 5:** Modification of the effect of gait speed on depressive symptoms by adherence to the Fat and Meat dietary pattern.

	**Adherence to the Fat and Meat Dietary Pattern**						**RRs (95%CI) for moderate adherence within gait speed strata**	**RRs (95%CI) for low adherence within gait speed strata**
	**High**		**Moderate**		**Low**			
	***N*** **cases/controls**	**RR (95% CI)**	***N*** **cases/controls**	**RR (95% CI)**	***N*** **cases/controls**	**RR (95% CI)**		
**Gait speed**								
Fast	8/119	Reference	6/85	0.9 (0.3–3.2), *p* = 0.96	4/78	1.2 (0.4–3.5), *p* = 0.81	0.9 (0.3–2.8), *p* = 0.78	1.0 (0.4–2.8), *p* = 0.96
Normal	51/481	2.0 (0.9–4.7), *p* = 0.11	63/469	2.1 (0.9–4.7), *p* = 0.11	65/474	1.7 (0.7–3.9), *p* = 0.26	1.0 (0.8–1.4), *p* = 0.98	0.9 (0.6–1.2), *p* = 0.22
Slow	7/103	3.4 (1.3–8.7), *p* = 0.02	5/86	3.2 (1.3–8.2), *p* = 0.02	6/93	1.9 (0.7–5.5), *p* = 0.29	0.9 (0.6–1.9), *p* = 0.93	0.7 (0.4–1.5), *p* = 0.35
RRs (95%CI) for normal gait within dietary pattern strata		1.9 (0.9–4.5), *p* = 0.12		2.2 (1–5.2), *p* = 0.09		1.5 (0.7–3.2), *p* = 0.34		
RRs (95%CI) for slow gait within dietary pattern strata		3.1 (1.3–7.6), *p* = 0.02		3.2 (1.2–8.1), *p* = 0.02		2.0 (0.8–5.4), *p* = 0.20		
Measure of interaction on the additive scale: RERI_low and slow_ (95%CI)			−2.5 (−4.1 to −0.8); *p* < 0.01				
Measure of interaction on the multiplicative scale: Ratio of RR_high and slow_ (95%CI)			0.3 (0.2–0.3); *p* < 0.01				

RRs were adjusted for sex, BMI, educational background, living alone, smoking and drinking habits, exercise habits, IADL score, hypertension status, diabetes status, hyperlipidemia status, and CVD status.

Moreover, low adherence to the Bread and Egg dietary pattern was associated with increased RRs of depressive symptoms in the slow walkers (2.3, 1.1–5.0, *p* = 0.04). Regarding high adherence to the Bread and Egg diet, the RR was substantially reduced to 1.1 (0.4–2.9, *p* = 0.91), and the associations were not significant (Table 6, Figure 2).

**Table 6:** Modification of the effect of gait speed on depressive symptoms by adherence to the Bread and Egg dietary pattern.

	**Adherence to the Bread and Egg dietary pattern**						**RRs (95%CI) for moderate adherence within**	**RRs (95%CI) for low adherence within**
	**High**		**Moderate**		**Low**			
	***N*** **cases/controls**	**RR (95%CI)**	***N*** **cases/controls**	**RR (95%CI)**	***N*** **cases/controls**	**RR (95%CI)**	**Gait speed strata**	**Gait speed strata**
**Gait speed**								
Fast	3/80	Reference	5/99	0.5 (0.2–1.5), *p* = 0.19	10/103	0.4 (0.1–1.3), *p* = 0.12	0.6 (0.2–1.5), *p* = 0.22	0.4(0.2–1.5), *p* = 0.15
Normal	64/478	1.2 (0.6–2.2), *p* = 0.77	58/473	1.1 (0.6–2.2), *p* = 0.79	57/473	1.2 (0.6–2.3), *p* = 0.7	1 (0.7–1.4), *p* = 0.93	1.1 (0.8–1.5), *p* = 1
Slow	21/71	1.1 (0.4–2.9), *p* = 0.91	11/57	1.6 (0.7–3.7), *p* = 0.34	7/53	2.3 (1.1–5.0), *p* = 0.04	1.3 (0.6–3.1), *p* = 0.57	1.9 (0.9–4.2), *p* = 0.11
RRs (95%CI) for normal gait within dietary pattern strata		1.1 (0.6–2.1), *p* = 0.82		2.3 (1–5.7.0), *p* = 0.08		3.4 (1.1–10.7), *p* = 0.04		
RRs (95%CI) for slow gait within dietary pattern strata		1.0 (0.5–2.5), *p* = 1.00		3.1 (1.1–8.6), *p* = 0.04		7.3 (2.2–24.2), *p* = 0.01		
Measure of interaction on the additive scale: RERI_low and slow_ (95%CI)			1.1 (−0.3–2.5); *p* = 0.12			
Measure of interaction on the multiplicative scale: Ratio of RR_low and slow_ (95%CI)			1.9 (0.2–18.2); *p* = 0.56			

RRs were adjusted for sex, BMI, educational background, living alone, smoking and drinking habits, exercise habits, IADL score, hypertension status, diabetes status, hyperlipidemia status, and CVD status.

## 4 Discussion

In this 6-year cohort study of 1,887 older adults aged 64–65 at baseline, slower gait speed was significantly associated with an increased risk of developing depressive symptoms by age 70/71. Importantly, our findings suggested that dietary patterns may influence the psychological impact of mobility decline. Specifically, the individuals with slower gait speed who adhered to a vegetable-rich or Bread and Egg dietary pattern tended to have a lower risk of depressive symptoms, although the interaction between gait speed and these dietary patterns was not statistically significant. Notably, a significant negative interaction was observed between slow gait and low adherence to a Fat and Meat dietary pattern, indicating that the individuals with slow gait who consumed less Fat and Meat had a substantially lower risk than would be expected under an additive model. To the best of our knowledge, this is the first study to investigate whether different dietary patterns can shape or moderate the mental health consequences of slow gait speed in older adults.

Associations between gait speed and depression have been widely reported in observational studies, with evidence suggesting that older adults who exhibit slower walking speed tend to experience more severe depressive symptoms (5). Although the relationship has often been described as bidirectional (27), a 16-year prospective cohort study found that slower gait speed was more likely to precede incident depression in men, rather than result from it (28). This temporal directionality was further supported by a meta-analysis of 13 longitudinal studies, which confirmed that reduced gait speed is a significant predictor of subsequent depressive symptoms (7). Building on this evidence, our study examined this association in a well-defined, age-specific cohort of 65-year-old adults and found that those with subjectively slower gait speed had nearly a threefold increased risk of developing depressive symptoms over a 6-year period.

Several mechanisms may underlie the observed association between slower gait speed and the onset of depressive symptoms. First, mobility impairments often lead to reduced physical activity, which is a well-established risk factor for depression through both behavioral and physiological pathways (29). Second, diminished gait performance may limit social participation and engagement in community activities, thereby reducing access to emotional and instrumental social support, both of which are well-documented protective factors against depression (30). Finally, from a neurobiological standpoint, gait disturbances and depressive symptoms may share common underlying pathophysiological mechanisms. For example, lesions in the prefrontal cortex and the presence of white matter hyperintensities have been linked to both impaired motor coordination and emotional dysregulation (31, 32). This overlap suggests that gait speed may serve as an early marker of broader neurodegenerative processes that also contribute to the development of late-life depression. Given their shared neurobiological basis, lifestyle factors such as diet may play a role in modifying these pathways.

To date, little is known about whether the association between slower gait speed and incident depressive symptoms varies across different dietary patterns. Diet is widely recognized as a modifiable lifestyle factor in the prevention of late-life depression (33). A meta-analysis of six randomized controlled trials found that dietary interventions significantly reduced the risk of depressive symptoms (34). Adherence to a general healthy diet and the Mediterranean diet was shown to be associated with lower depressive symptom scores over time (35). Our study examined the interaction between dietary patterns and gait speed on the risk of depressive symptoms. We found a dose–response relationship, indicating that higher adherence to either a vegetable-rich or a Bread and Egg dietary pattern was associated with a progressively lower risk of depressive symptoms among older adults with mobility decline. These results may reflect the existence of distinct pathways linking gait speed to depressive symptoms. The vegetable-based dietary pattern, rich in antioxidants, fiber, and polyphenols, has been associated with lower levels of systemic inflammation, as indicated by biomarkers such as C-reactive protein and interleukin 6, both of which are implicated in depression and age-related physical decline (36–38). Second, the Bread and Egg dietary pattern, which may be higher in protein and calcium, could support muscle strength and physical function (39–41), subsequently reducing social isolation, which is also a known risk factor for depression (42).

In contrast, our findings revealed a significant negative interaction between slow gait speed and low adherence to a dietary pattern characterized by high intake of Fat and Meat, indicating that reduced consumption of these foods may attenuate the psychological risks associated with mobility decline. Diets rich in saturated fats, red meat, and processed meat, which typically define a Western dietary pattern, have been consistently linked to adverse mental health outcomes, including depressive symptoms and anxiety disorders, in observational studies and meta-analyses (43, 44). Multiple biological mechanisms may underlie this relationship. For instance, high consumption of saturated fats and processed meats promotes chronic systemic inflammation and oxidative stress, which are critical pathways implicated in the pathogenesis of depression (45). In addition, diets high in saturated fat may negatively affect hippocampal integrity by impairing neurogenesis and synaptic plasticity and by promoting neuronal inflammation, thereby exacerbating the neurobiological vulnerabilities commonly associated with mobility impairment (46, 47). Moreover, excessive meat intake has been associated with alterations in gut microbiome composition and function, potentially disrupting the gut–brain axis and further contributing to mood dysregulation (48, 49). Therefore, adopting a dietary approach that reduces the intake of fats and meats may confer neuroprotective benefits, particularly for older adults with pre-existing mobility limitations, by minimizing exposure to these detrimental pathways. While functional performance is influenced by a variety of factors, our findings highlight diet as a key modifiable component within this multifactorial context.

These findings not only advance our understanding of the interplay between diet and physical function in mental health but also point to actionable opportunities for early intervention. Dietary modification may be more feasible and acceptable than exercise-based programs among older adults with mobility limitations, who often experience musculoskeletal pain or fatigue (50). Identifying slow gait speed as a simple, non-invasive marker enables the timely implementation of preventive strategies before the onset of clinically significant depression. Therefore, incorporating dietary counseling into routine geriatric care may represent a scalable and cost-effective approach to addressing both physical and mental health challenges in aging populations.

This study has several strengths, including its large, community-based cohort, prospective design, and evaluation of the interaction effects between gait speed and dietary patterns on depressive symptoms. However, some limitations should be noted. First, subjective gait speed was self-reported rather than objectively measured, which may have introduced misclassification bias. However, the validity of subjective gait speed in this database was verified in a previous study (51). Second, although the GDS-15 is widely used in both clinical and research settings and has demonstrated good sensitivity and specificity in older populations, it is still possible that it may overestimate or underestimate the true prevalence of depressive episodes. Third, dietary intake was assessed using a self-reported FFQ, which is subject to recall bias and misreporting. Fourth, given the observational design, residual or unmeasured confounding—such as anxiety or orthorexia (52)—cannot be fully ruled out. Nonetheless, all reported estimates were derived from multivariable models with simultaneous adjustment for major risk factors. Moreover, the small number of participants in some extreme gait speed subgroups may limit the generalizability of the findings in these specific strata, although the overall results remained robust. Finally, the generalizability of our findings to populations from heterogeneous ethnic groups may be limited. The study was conducted in a homogeneous Japanese cohort, and dietary patterns, lifestyle factors, and cultural perceptions of mental health may differ substantially across populations.

In conclusion, our findings suggest that dietary patterns may moderate the association between slower gait speed and depressive symptoms in older adults. Specifically, we observed a significant negative interaction between slow gait speed and low adherence to a fat- and meat-heavy dietary pattern, indicating that reduced intake of such foods may attenuate the psychological risks associated with mobility decline. While trends toward a weaker association were also noted for higher adherence to vegetable-rich and Bread and Egg dietary patterns, these interactions did not reach statistical significance. Collectively, these results highlight the potential value of incorporating dietary assessment into clinical evaluations of older adults, particularly those with gait impairments. Further research is needed to elucidate the underlying mechanisms and to confirm these observational findings in interventional or experimental settings.

## Data Availability

The raw data supporting the conclusions of this article will be made available by the authors, without undue reservation.
